# Supplementary material for: Interferon-γ couples CD8+ T cell avidity and differentiation during infection
Source: Nat Commun. 2023 Oct 23;14:6727. doi: 10.1038/s41467-023-42455-4 (PMC10593754; doi:10.1038/s41467-023-42455-4)
Supplement: Supplementary file 1 — Supplementary Information [file 41467_2023_42455_MOESM1_ESM.pdf]

Interferon-γ couples CD8[+] T cell avidity and differentiation during infection

Lion F.K. Uhl, Han Cai, Sophia L. Oram, Jagdish N. Mahale, Andrew J. MacLean, Julie M. Mazet, Theo Piccirilli, Alexander J. He, Doreen Lau, Tim Elliott, and Audrey Gerard

Supplementary Figures

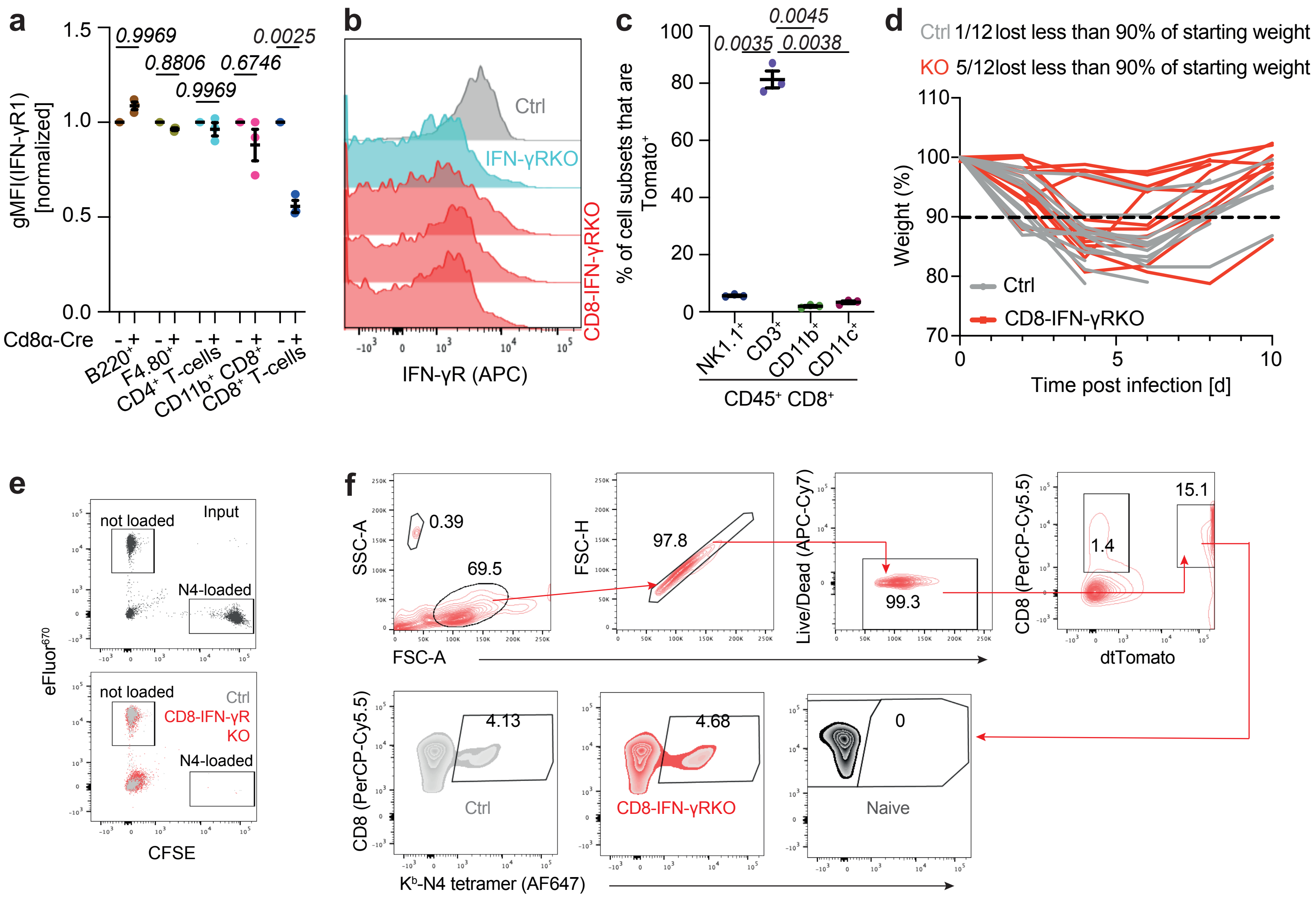

Figure S1: Specific deletion of IFN-γR in CD8⁺ T-cells.

(a-c) Cell-specific deletion of IFN-γR in different immune cell subtypes of CD8-IFN-γRKO (KO) or control (Ctrl) mice was assessed either by flow cytometry of IFN-γR1 expression **(a-b)** or tdTomato expression **(c)** (n = 3 animals). Data are representative of ≥ 2 independent experiments. **(d)** KO and Ctrl mice were infected with $4x10^4$ pfu X31-OVA and weight was measured every two days. Graph shows weight of individual mice over time. Data are from 3 independent experiments (n = 12 animals). **(e)** KO (red) and Ctrl (grey) mice were infected with LM-OVA and injected with ad-mixed N4-loaded or unloaded target splenocytes after 7 days to quantify in vivo cytotoxicity. Graph shows dotplot example of antigen-specific in vivo cytotoxicity (n = 8 animals). **(f)** Representative gating strategy for isolating endogenous OVA-specific, tetramer⁺ CD8⁺ T-cells. Data are from ≥3 independent experiments. Two-way ANOVA and Šidák's multiple comparison test **(a),** one-way ANOVA and Tukey's multiple comparison test **(c)**. Error bars indicate the mean ± s.e.m.

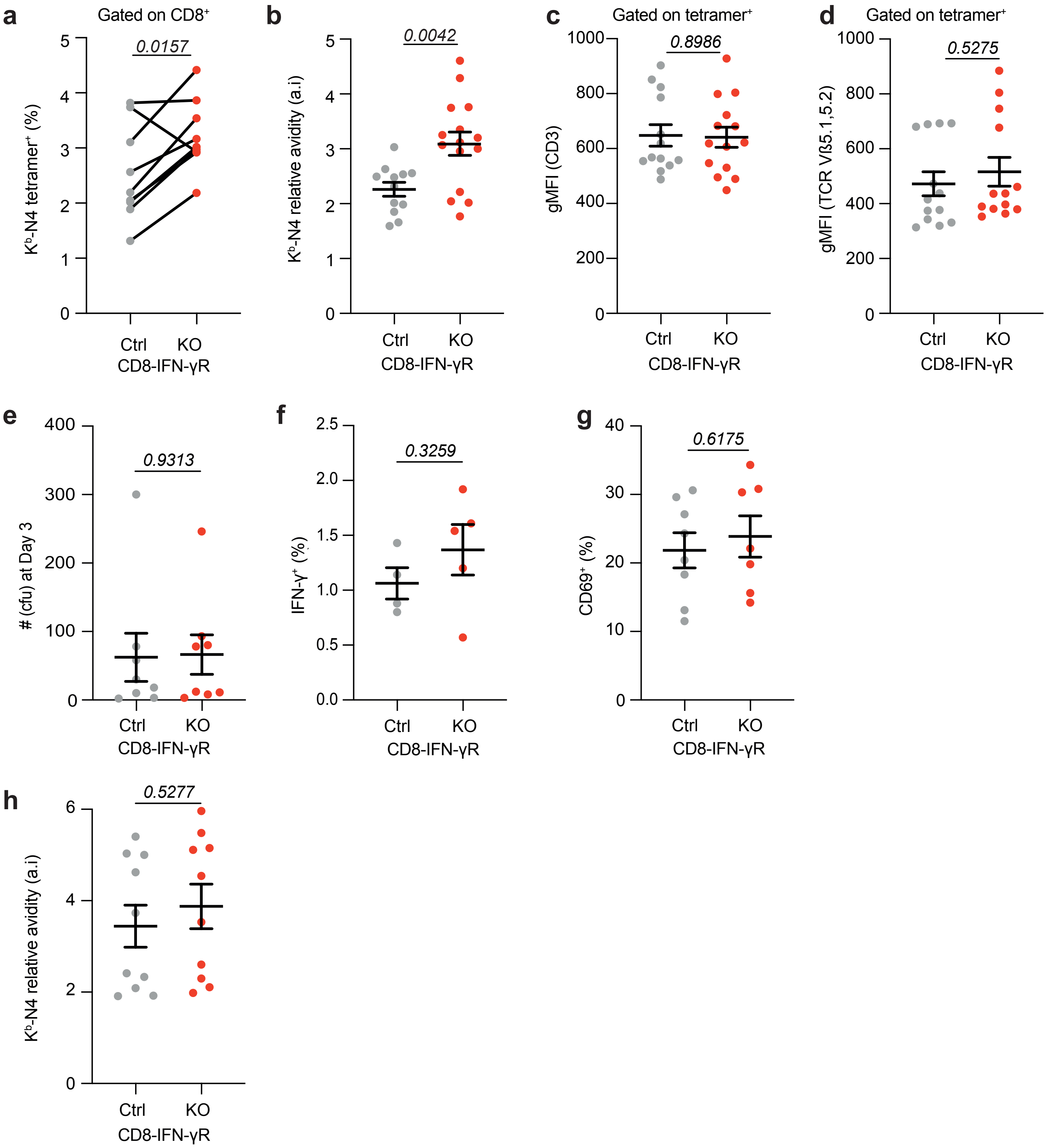

Figure S2: IFN-γR deletion in $CD8^+$ T-cells increases the avidity of the primary response. **(a-d)** CD8-IFN-γRKO (KO, red) and control (Ctrl, grey) mice were infected with LM-OVA. Spleens were isolated and N4-tetramer$^+$ $CD8^+$ T-cells were analyzed after 9 days. **(a)** Graph shows the average relative abundance of N4-tetramer$^+$ $CD8^+$ T-cells (n = 8 experiments, 3-5 mice per experiment). **(b)** Relative avidity of N4-tetramer$^+$ $CD8^+$ T-cells, calculated by dividing tetramer gMFI by CD3 gMFI (n = 12 Ctrl,15 KO animals). **(c)** Quantification of CD3 (n = 13 Ctrl,14 KO animals) and **(d)** TCR Vβ5.1,5.2 (n = 13 animals) expression of N4-tetramer$^+$ $CD8^+$ T-cells. **(e)** KO (grey) and Ctrl (red) mice were infected with LM-OVA. Spleens were isolated after 3 days to analyze the bacterial load (n = 8 animals). **(f-g)** KO (grey) and Ctrl (red) mice were infected with LM-OVA and spleens were isolated after 24h to analyze IFN-γ **(f)** (n = 4 Ctrl, 5 KO animals) and CD69 **(g)** (n = 7 Ctrl, 8 KO animals) expression by flow cytometry. **(h)** KO (grey) and Ctrl (red) mice were infected with LM-OVA and spleens were isolated after 5 days. Graph shows the relative avidity of N4-tetramer$^+$ $CD8^+$ T-cells (n = 10 animals). Data are from ≥3 independent experiments. Two-tailed paired **(a)** and unpaired **(b-h)** student's t-test. Error bars indicate the mean ± s.e.m.

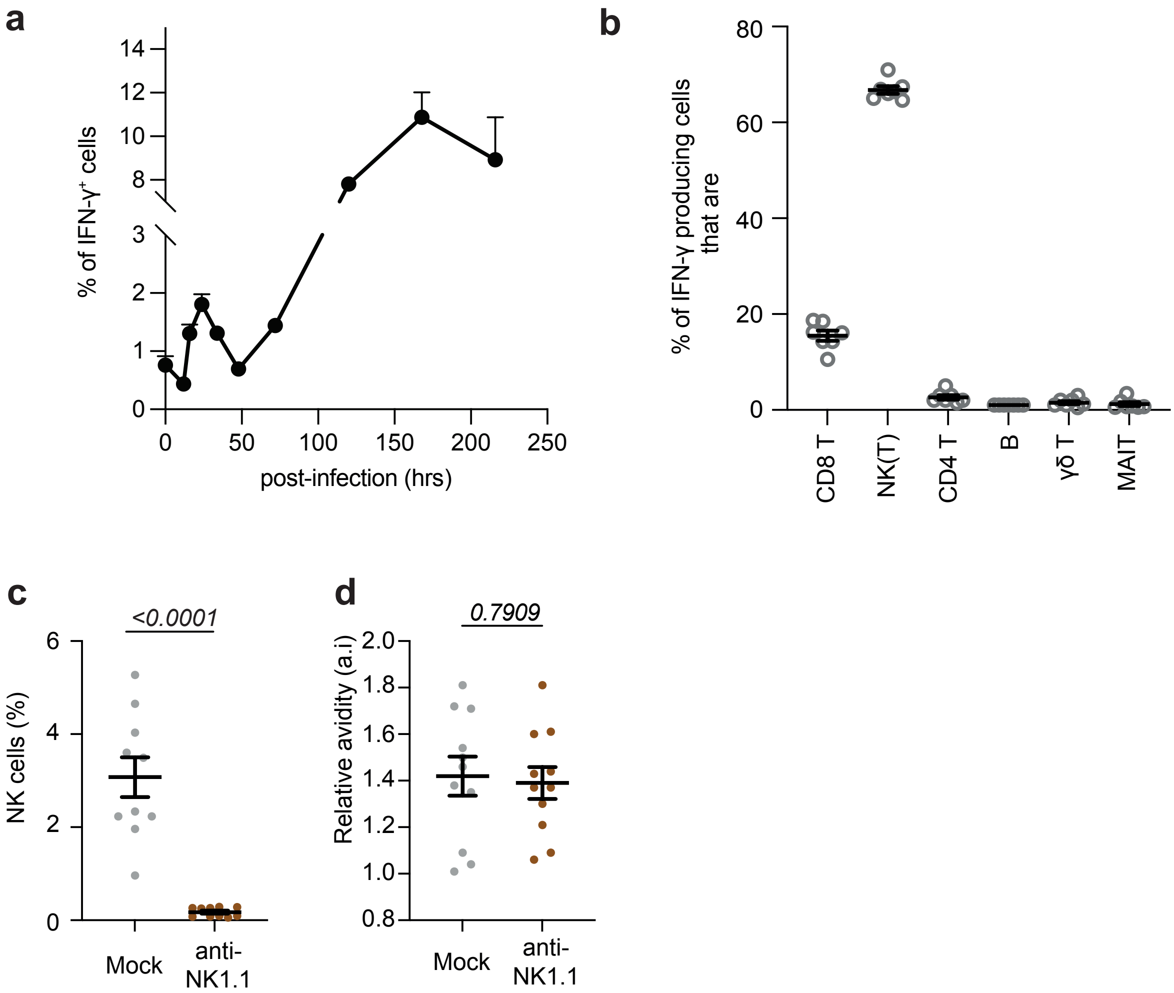

Figure S3: Immune cells producing IFN-γ during priming.
(a) GREAT mice were infected with LM-OVA and spleens were isolated when indicated to analyze IFN-γ (YFP)+ expression by flow cytometry (n = 3-9 animals). **(b)** GREAT mice were infected with LM-OVA and spleens were isolated after 24 hours to analyze the IFN-γ (YFP)+ expression of different immune cell subsets by flow cytometry (n = 7 animals). **(c-d)** WT mice were treated with depleting NK1.1 antibodies (brown) or control antibodies (grey) and infected with LM-OVA. Splenocytes were isolated after 9 days. Relative abundance of NK cells **(c)** (n = 10 animals) and relative avidity of N4-tetramer$^+$ CD8$^+$ T-cells **(d)** (n = 10 Mock, 11 anti-NK1.1 treated animals), analyzed by flow cytometry. Relative avidity was calculated by dividing tetramer MFI by CD3 MFI. Data are from ≥3 independent experiments. Two-tailed unpaired Student's t-test **(c-d)**. Error bars indicate the mean ± s.e.m.

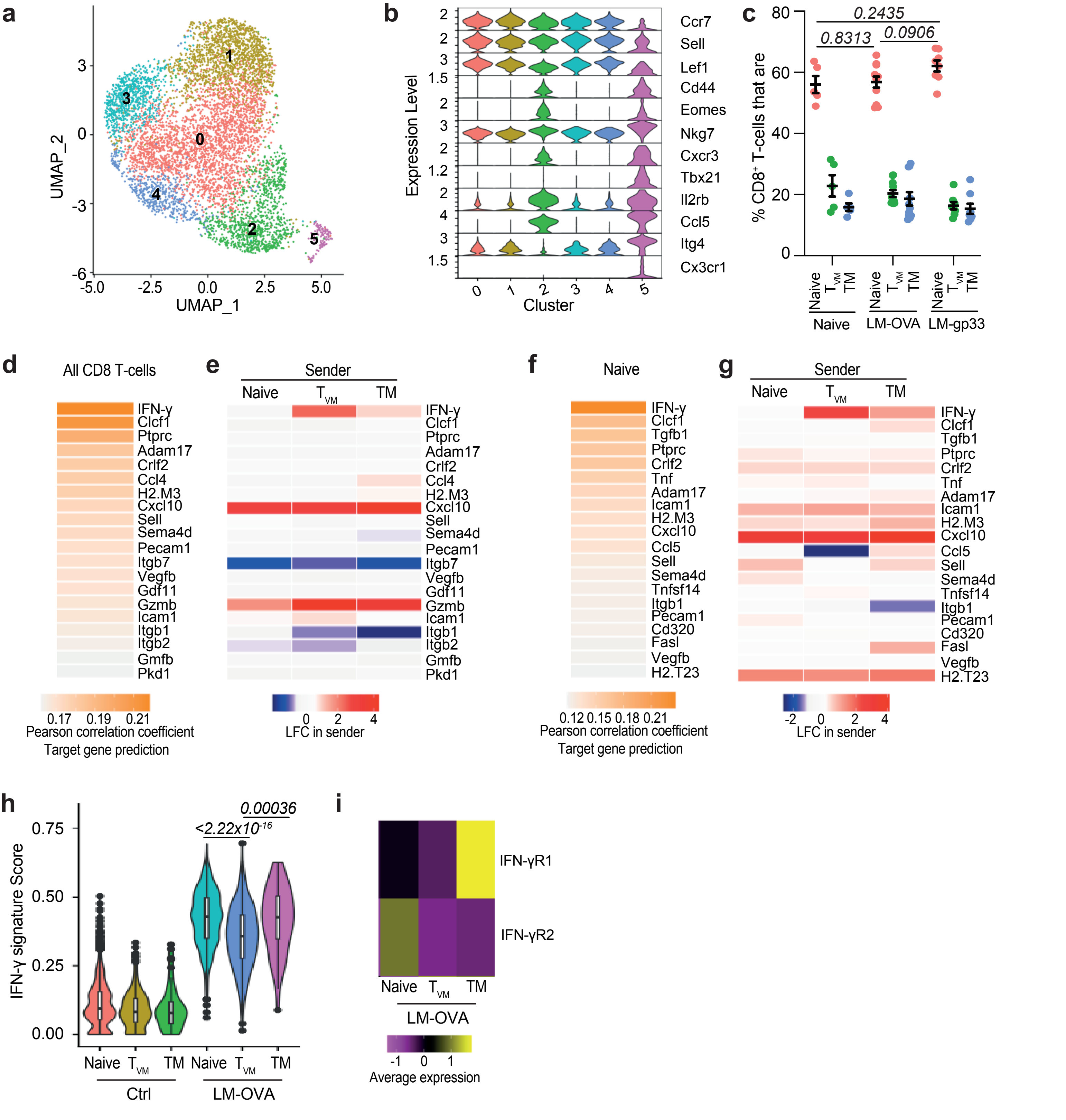

Figure S4: IFN-γ-sensing by CD8⁺ T-cells is paracrine and enabled by T_{VM}.

(a-b, d-i) CD8⁺ T-cells from naïve (Ctrl) or LM-OVA infected mice were sorted after 24h and subjected to scRNA-seq analysis (n = 3224 cells from 3 Ctrl mice, 3495 cells from 3 LM-OVA infected mice). **(a)** Graph-based clustering of the identified clusters. **(b)** ViolinPlot shows the expression of selected markers. **(c)** GREAT mice were infected with either LM-OVA or -gp33 and the relative abundance of naïve (red), T_{VM} (green) or true memory (TM, blue) CD8⁺ T-cells among CD8⁺ T-cells were analyzed by flow cytometry 24h after infection (n = 8 animals from naive condition, 11 from other conditions). Two-way ANOVA and Šidák's multiple comparison test. **(d-g)** NicheNet analysis of scRNA-seq. **(d,f)** Ligand activity prediction, ordered by Pearson correlation coefficient between regulatory potential scores in all **(d)** or naïve **(f)** CD8⁺ T-cells. **(e,g)** Average ligand expression in the "sender" cells when all **(e)** or naïve **(g)** CD8⁺ T-cells were selected as receivers. **(h)** The violin plots show the expression score (y axes) of the IFN-γ signaling gene signature in each of the identified clusters (x axes). Box plots indicate median (middle line), 25th, 75th percentile (box). Two-tailed Wilcoxon rank sum test. **(i)** Heatmap shows the relative average expression of IFNγR1 and IFNγR2 in the different cell clusters.

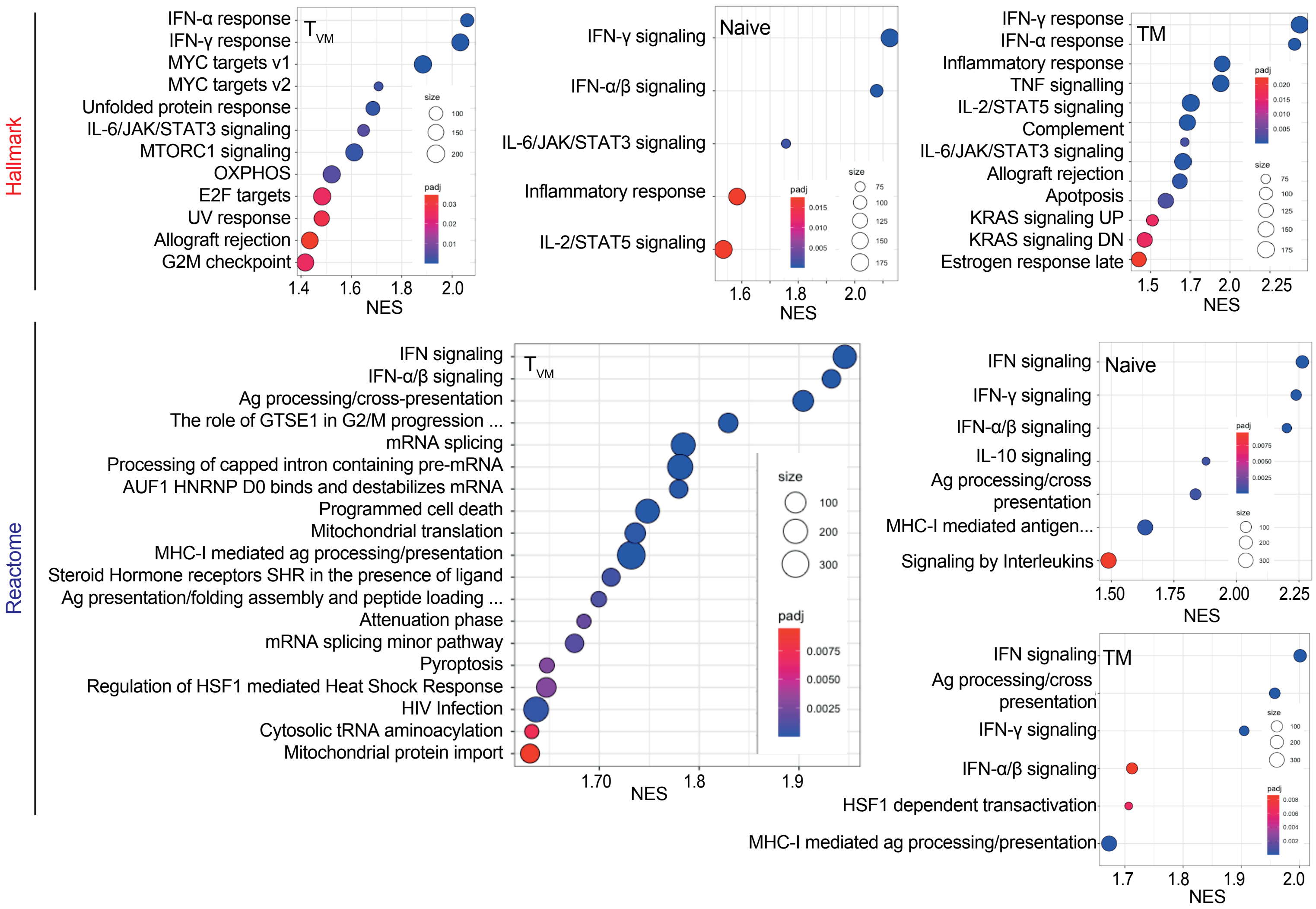

Figure S5: Pathways elicited by LM-OVA in CD8⁺ T cells during priming.
CD8⁺ T-cells from naïve (Ctrl) or LM-OVA infected mice were sorted after 24h and subjected to scRNA-seq analysis (n = 3224 cells from 3 Ctrl mice, 3495 cells from 3 LM-OVA infected mice). Cells were clustered as in Figure S4. Pathway analysis between control and LM-OVA infected samples for TM, T_{VM} or naïve CD8⁺ T-cells. Graphs show the Normalized Enrichment Score (NES) for the Hallmark pathways (top panels) and Reactome pathways (bottom panels).

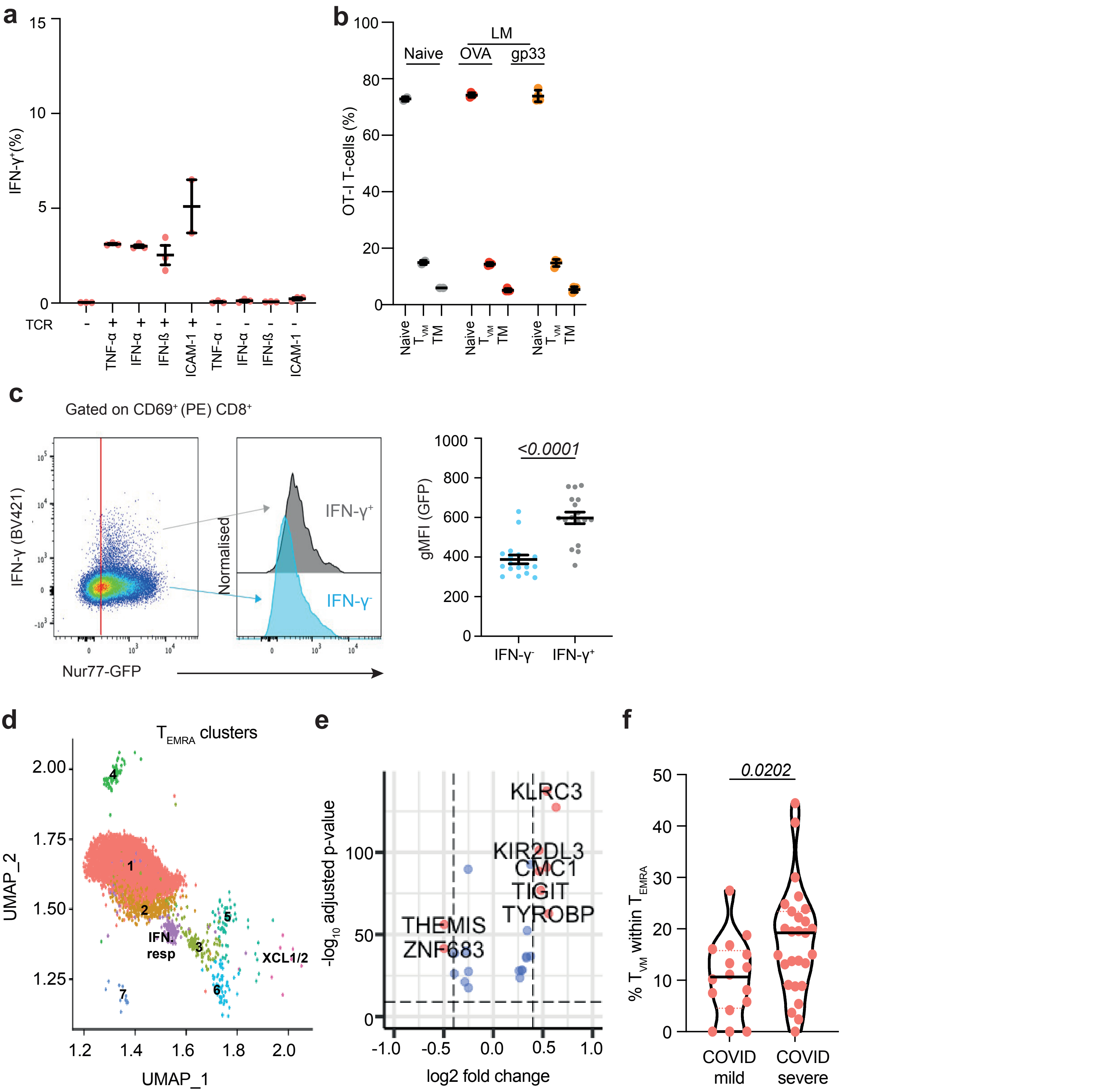

Figure S6: Regulation of IFN-γ-production by CD8⁺ T-cells during priming.
(a) CD8⁺ T-cells were stimulated in vitro with TCR/CD28 antibodies, IL-12, IL-18, ICAM-1, TNF, IL-15 or Type I IFN as indicated. IFN-γ expression was analyzed by flow cytometry after 24h (n = 3 animals). **(b)** WT mice were transferred with $2x10^6$ GREAT OT-I CD8⁺ T-cells the relative abundance of naïve, T_{VM} or TM CD8⁺ T-cells among OT-I splenocytes was analyzed by flow cytometry 24h post-infection with LM-OVA (red) or LM-gp33 (orange) (n = 2-4 animals). **(c)** Nur77-GFP mice were infected with LM-OVA and Nur77-GFP and IFN-γ expression was evaluated by flow cytometry 24h post-infection. Representative flow plot (left panel) and histograms (middle panel) of Nur77-GFP and IFN-γ expression. Quantification (right panel) of Nur77-GFP expression in IFN-γ^{neg} and IFN-γ^{pos} CD8⁺ T-cells (n = 17 animals). Two-tailed unpaired Student's t-test. **(d-f)** TEMRA clusters from the COMBAT human blood atlas were reanalyzed according to COVID-19 severity. **(d)** Graph-based clustering of the TEMRA clusters. **(e)** Volcanoplot shows specific differentially regulated genes between T_{VM} and other TEMRA. Green dots: genes with log2 (fold-change) value >0.5 or <-05; blue dots: genes with an adjusted p value<0.05; red dots: genes with log2 (fold-change) value >0.5 or <-05 and an adjusted p value<0.05. Wilcoxon rank sum test. **(f)** Violinplot shows the percentage of TEMRA that are T_{VM} according to disease state and severity. Two-sided Wilcoxon signed-rank test (COVID mild = 12, COVID severe = 25 patients). **(a-c)** Data are representative of≥3 independent experiments. Error bars indicate the mean ± s.e.m.

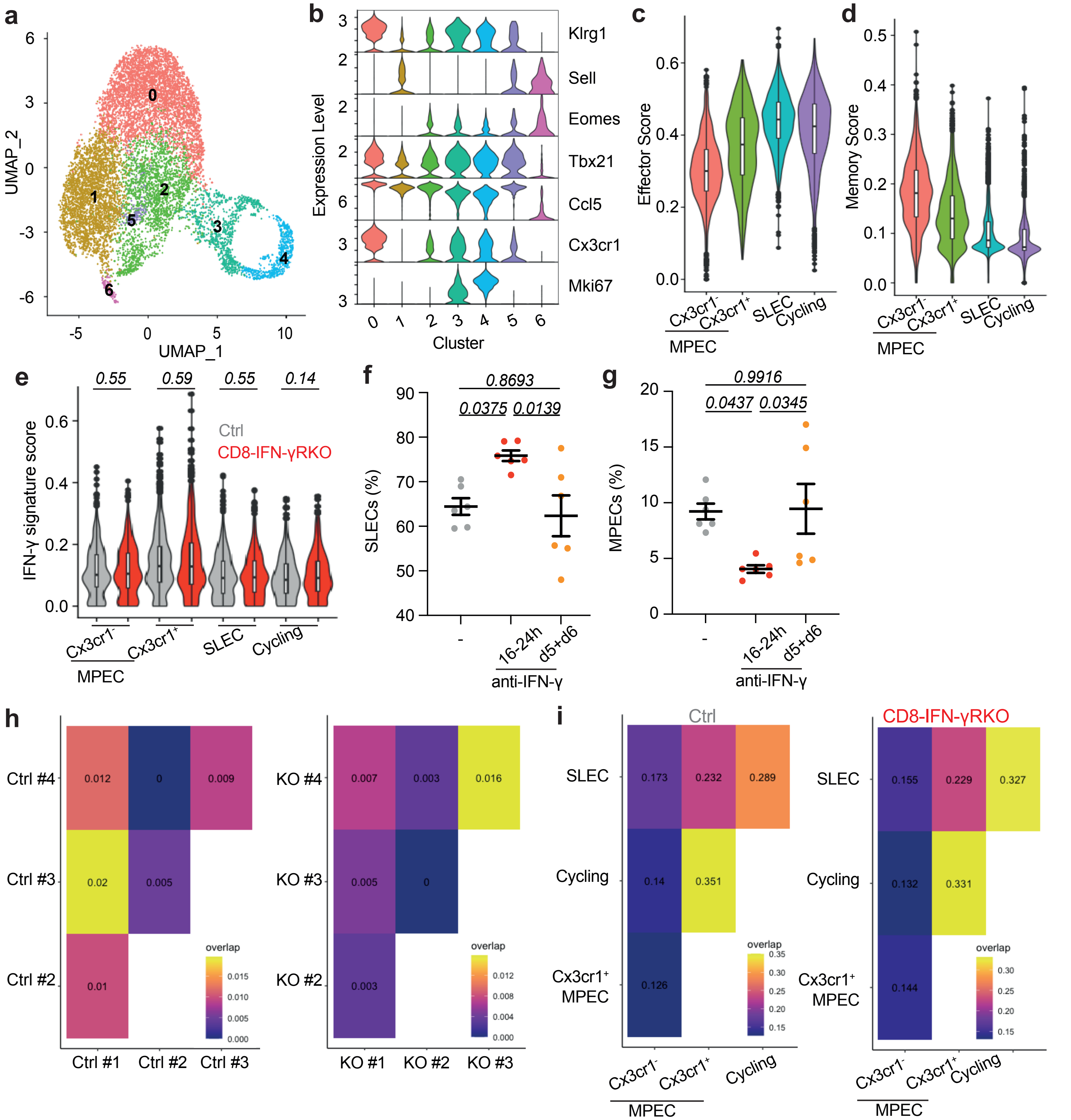

Figure S7: IFN-γ-sensing by CD8⁺ T-cells does not regulate their intrinsic differentiation.
(a-e, h-k) CD8-IFN-γRKO (KO) and control (Ctrl) mice were infected with LM-OVA, N4-tetramer^{+} CD8^{+} T-cells were sorted from spleens 9 days post-infection and subjected to scRNA-seq and scTCR-seq analysis (n = 5646 Ctrl; 4837 KO cells from 4 animals). **(a)** Graph-based clustering of initial clusters. **(b)** ViolinPlot shows the expression of selected markers. **(c-e)** The violin plots show the expression score (y axes) of an effector **(c)**, memory **(d)** and IFN-γ signaling **(e)** gene signature in each of the identified clusters (x axes). Box plots indicate median (middle line), 25th, 75th percentile (box). Pairwise comparisons two-sided Wilcoxon signed-rank test. **(f-g)** WT mice were infected with LM-OVA and mice were either left untreated (grey) or treated with anti-IFN-γ 16-24 hours (red) or at day 5 and 6 (orange) post-infection. Splenocytes were isolated 9 days post infection and cell subsets were analyzed by flow cytometry using the surface markers KLRG1 and CD127. Graphs show the relative proportion of SLECs **(f)** (KLRG1+ CD127-), and MPECs **(g)** (KLRG1- CD127+) (n = 6 animals) of N4-tetramer^{+} CD8^{+} T-cells. Data are from 2 independent experiments. One-way ANOVA and Tukey's multiple comparison test. Error bars indicate the mean ± s.e.m. **(h)** Frequency of TCR overlap between mice from combined scRNA- and scTCR-seq. **(i)** Frequency of TCR overlap between clusters from combined scRNA- and scTCR-seq.

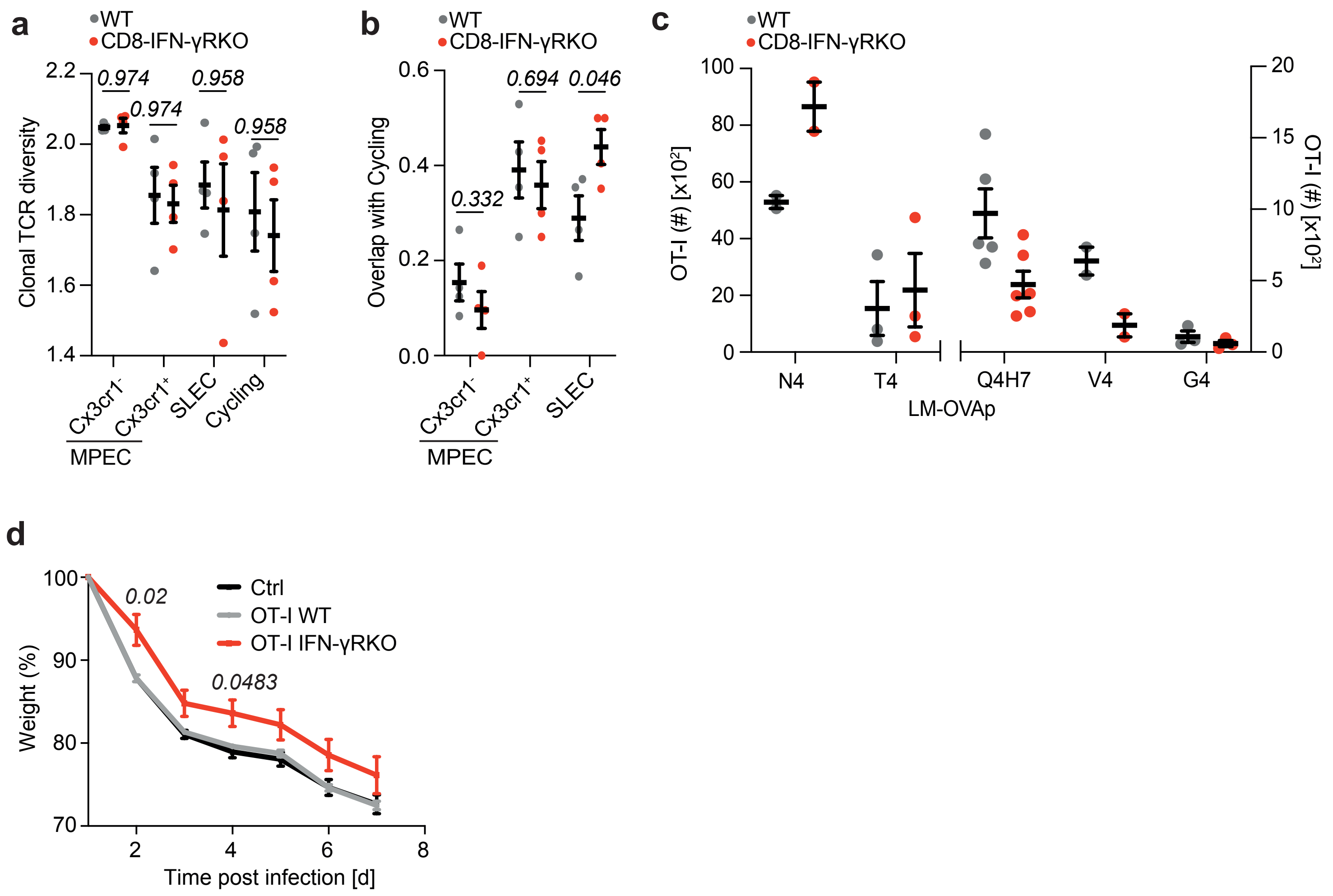

Figure S8: IFN-γ-sensing differentially impacts high- and low-avidity CD8⁺ T-cells.
(a-b) CD8-IFN-γRKO (KO, red) and control (Ctrl, grey) mice were infected with LM-OVA, N4-tetramer⁺ CD8⁺ T-cells were sorted from spleens 9 days post-infection and subjected to scRNA-seq and scTCR-seq analysis (n = 5646 Ctrl; 4837 KO cells from 4 animals). Cell clusters are identified as in Figure 5. Data was subsetted on the four most represented TCR Vb chains. **(a)** Relative TCR clonal diversity (Shannon index) by cell state and genotype extracted from scTCR-seq analysis. **(b)** Clonal overlap between the cycling cluster and the other clusters from scTCR-seq analysis. **(c)** WT mice were transferred with 50.000 ad-mixed WT (grey) and IFN-γRKO (red) OT-I T-cells and infected with LM expressing the indicated OVA peptides. Graph shows the absolute OT-I numbers, quantified by flow cytometry 9 days post-infection (n = 6 animals). **(d)** Mice were either transferred with WT (grey) or IFN-γRKO (red) OT-I T-cells and infected with 10^5 pfu of X31-OVA. Weight was measured every day (n = 7 animals). Data are from ≥3 independent experiments. Two-way ANOVA and Šidák's multiple comparison test **(a-b)**. Error bars indicate the mean ± s.e.m.
