# Supplementary material for: Interferon-γ couples CD8+ T cell avidity and differentiation during infection
Source: Nat Commun. 2023 Oct 23;14:6727. doi: 10.1038/s41467-023-42455-4 (PMC10593754; doi:10.1038/s41467-023-42455-4)
Supplement: Supplementary file 3 — Reporting Summary [file 41467_2023_42455_MOESM3_ESM.pdf]

nature portfolio

Corresponding author(s): Audrey Gerard

Last updated by author(s): 2023/09/27

Reporting Summary

Nature Portfolio wishes to improve the reproducibility of the work that we publish. This form provides structure for consistency and transparency in reporting. For further information on Nature Portfolio policies, see our Editorial Policies and the Editorial Policy Checklist.

Statistics

For all statistical analyses, confirm that the following items are present in the figure legend, table legend, main text, or Methods section.

n/a	Confirmed	
☐	☒	The exact sample size (n) for each experimental group/condition, given as a discrete number and unit of measurement
☐	☒	A statement on whether measurements were taken from distinct samples or whether the same sample was measured repeatedly
☐	☒	The statistical test(s) used AND whether they are one- or two-sided<br>*Only common tests should be described solely by name; describe more complex techniques in the Methods section.*
☒	☐	A description of all covariates tested
☐	☒	A description of any assumptions or corrections, such as tests of normality and adjustment for multiple comparisons
☐	☒	A full description of the statistical parameters including central tendency (e.g. means) or other basic estimates (e.g. regression coefficient) AND variation (e.g. standard deviation) or associated estimates of uncertainty (e.g. confidence intervals)
☐	☒	For null hypothesis testing, the test statistic (e.g. F, t, r) with confidence intervals, effect sizes, degrees of freedom and P value noted<br>*Give P values as exact values whenever suitable.*
☒	☐	For Bayesian analysis, information on the choice of priors and Markov chain Monte Carlo settings
☒	☐	For hierarchical and complex designs, identification of the appropriate level for tests and full reporting of outcomes
☐	☒	Estimates of effect sizes (e.g. Cohen's d, Pearson's r), indicating how they were calculated

Our web collection on statistics for biologists contains articles on many of the points above.

Software and code

Policy information about availability of computer code

Data collection	Flow cytometry data was acquired using BDFACSDiva (v8.0) software<br>No new bioinformatics tools or algorithms were created
Data analysis	Flow cytometry data was analyzed using Flowjo (v10.4.2).<br>Single cell RNA sequencing data was analyzed using R (v 4.1.3), Seurat (v4.0.6), UCell (v.1.3), fgsea, NicheNet, scRepertoire (v.1.1.4) .<br>Data was visualized using Graphpad (V8.4.1, Prism software), EnhancedVolcano (v1.12.0), GGplot2 (v3.3.5) and ggpubr (v0.5.0).

For manuscripts utilizing custom algorithms or software that are central to the research but not yet described in published literature, software must be made available to editors and reviewers. We strongly encourage code deposition in a community repository (e.g. GitHub). See the Nature Portfolio guidelines for submitting code & software for further information.

Data

Policy information about availability of data

All manuscripts must include a data availability statement. This statement should provide the following information, where applicable:

- Accession codes, unique identifiers, or web links for publicly available datasets
- A description of any restrictions on data availability
- For clinical datasets or third party data, please ensure that the statement adheres to our policy

The mouse scRNAseq and scTCRseq data generated in this study have been deposited in the GEO database under accession code GSE244203. Datasets reused in

this study: EGAS0000100549344. All data are included in the Supplemental Information or available from the authors upon reasonable requests, as are unique reagents used in this Article. The raw numbers for charts and graphs are available in the Source Data file whenever possible. Source data are provided with this paper.

Research involving human participants, their data, or biological material

Policy information about studies with human participants or human data. See also policy information about sex, gender (identity/presentation), and sexual orientation and race, ethnicity and racism.

Reporting on sex and gender	n/a
Reporting on race, ethnicity, or other socially relevant groupings	n/a
Population characteristics	n/a
Recruitment	n/a
Ethics oversight	n/a

Note that full information on the approval of the study protocol must also be provided in the manuscript.

Field-specific reporting

Please select the one below that is the best fit for your research. If you are not sure, read the appropriate sections before making your selection.

[x] Life sciences [ ] Behavioural & social sciences [ ] Ecological, evolutionary & environmental sciences

For a reference copy of the document with all sections, see nature.com/documents/nr-reporting-summary-flat.pdf

Life sciences study design

All studies must disclose on these points even when the disclosure is negative.

Sample size	Sample size was estimated based on prior experience and complexity. Whenever possible, we used g*power to calculate sample size. We assumed that a difference of at least 20% would be necessary to be biologically significant. Given a high variation, we calculated that at least 5 mice per group were necessary.
Data exclusions	no exclusion
Replication	All findings were replicated in two to 20 separate experiments (as specified in legends), with the exception of the single cell RNA sequencing experiment, in which data was aggregated from multiple separate biological samples as indicated in the figure legends and methods section.<br>All separate experiments yielded comparable trends and results.
Randomization	For mouse experiments involving multiple genotypes, we used littermates, and controlled that gender and age was similar between all groups.<br>Whenever possible, comparison was done between populations within the same mouse, for which randomization is not required.<br>For experiments not involving mouse tissues randomization was not required as samples were fixed and therefore not impacted by the time at which they were processed.
Blinding	No blinding was performed during mouse experiments when all mice within an experiment received identical treatments. No subjective scoring methods which would require blinding were used. Most experiments were performed and repeated independently by two researchers.<br>Flow cytometry data were collected in an automatic and unbiased manner.

Reporting for specific materials, systems and methods

We require information from authors about some types of materials, experimental systems and methods used in many studies. Here, indicate whether each material, system or method listed is relevant to your study. If you are not sure if a list item applies to your research, read the appropriate section before selecting a response.

Materials & experimental systems

n/a	Involved in the study
☐	☒ Antibodies
☒	☐ Eukaryotic cell lines
☒	☐ Palaeontology and archaeology
☐	☒ Animals and other organisms
☒	☐ Clinical data
☒	☐ Dual use research of concern
☒	☐ Plants

Methods

n/a	Involved in the study
☒	☐ ChIP-seq
☐	☒ Flow cytometry
☒	☐ MRI-based neuroimaging

Antibodies

Antibodies used	anti-CD8 (Biolegend, clone: 53-6.7), anti-CD4 (Biolegend, clone: RM4-5), anti-CD69 (Biolegend, clone: H1.2F3), anti-CD44 (Biolegend, clone: IM7), anti-CD49d (Biolegend, clone: R1-2), anti-NK1.1 (Biolegend, clone: PK137), anti-KLRGI (Biolegend, clone: 2F1/KLRF1). Antibodies used for intracellular cytokine staining included anti-IFN-gamma (Biolegend, clone: XMG1.2) and anti-TNF (Biolegend, clone: MP6-XT22). For tetramer staining: Alexa Fluor 647- or BV421-conjugated, N4-specific MHC I tetramers (National Institutes of Health Tetramer Core Facility [Emory University, Atlanta])
Validation	All antibodies were obtained from commercial vendors and we based specificity on descriptions and information provided in corresponding their Data Sheet. In addition, we used isotype controls, KO cells (or other cells that we know did not express the protein of interest) to validate our staining.<br>Dilution optimization was performed on splenocytes.<br><br>Biolegend validation-Flow Cytometry Reagents: Specificity testing of 1-3 target cell types with either single- or multi- color analysis (including positive and negative cell types). Once specificity is confirmed, each new lot must perform with similar intensity to the in-date reference lot. Brightness (MFI)i s evaluated from both positive and negative populations. Each lot product is validated by QC testing with a series of titration dilutions. https://www.biolegend.com/en-us/quality/quality-control

Animals and other research organisms

Policy information about studies involving animals; ARRIVE guidelines recommended for reporting animal research, and Sex and Gender in Research

Laboratory animals	species: mus musculus,<br>strains: C57bl/6, CD45.1, OT-I, CD8a-Cre, IFNgRflox (CD119flox), ROSA-Tomato, IFNgRKO, GREAT, Nur77-GFP<br>sex: male and female<br>age: 6-14 weeks
Wild animals	study did not involve wild animals
Reporting on sex	Both genders were used in experiments. Males and Females were equally distributed throughout conditions. We did not initially find any difference between males and females and therefore did not take gender into account for analysis.
Field-collected samples	study did not involve field-collected samples
Ethics oversight	All experiments involving mice were conducted in agreement with the United Kingdom Animal (Scientific Procedures) Act of 1986 and performed in accordance to approved experimental procedures by the Home Office and the Local Ethics Reviews Committee (University of Oxford).

Note that full information on the approval of the study protocol must also be provided in the manuscript.

Flow Cytometry

Plots

Confirm that:

☒ The axis labels state the marker and fluorochrome used (e.g. CD4-FITC).

☒ The axis scales are clearly visible. Include numbers along axes only for bottom left plot of group (a 'group' is an analysis of identical markers).

☐ All plots are contour plots with outliers or pseudocolor plots.

☒ A numerical value for number of cells or percentage (with statistics) is provided.

Methodology

Sample preparation	See method section of manuscript
Instrument	FACSAria™ II (BD) was used for sorting and Fortessa X-20 for analysis
Software	Data collection: BDFACSDiva (v8.0) software<br>Data analysis: Flowjo v10.8.1
Cell population abundance	Populations were sorted at >95% purity, determined by flow cytometric analysis of post-sort samples
Gating strategy	Cells were sorted based on expression of N4-tetramer, CD3, CD8, and Tomato.

☒ Tick this box to confirm that a figure exemplifying the gating strategy is provided in the Supplementary Information.
