# Supplementary material for: Interferon-γ couples CD8+ T cell avidity and differentiation during infection
Source: Nat Commun. 2023 Oct 23;14:6727. doi: 10.1038/s41467-023-42455-4 (PMC10593754; doi:10.1038/s41467-023-42455-4)
Supplement: Supplementary file 4 — Description of Additional Supplementary Files [file 41467_2023_42455_MOESM4_ESM.pdf]

Title: Supplementary Dataset 1:
Description: Differentially expressed genes between the different TEMRA clusters from the COMBAT dataset. List of differentially expressed genes between TVM and other TEMRA populations. Significant differentially expressed genes between clusters were identified using the "FindAllMarkers" function, Wilcoxon test and selecting markers expressed in at least 25% of cells.

Title: Supplementary Dataset 2:
Description: Gene signatures used in this study. List of genes to compute the signatures used in this study.

Title: Supplementary Dataset 3:
Description: Antibodies used in this study
